# Computational analysis of intracardiac collision risk and optimal site for right ventricular leadless left bundle branch area pacing: A simulation study

**DOI:** 10.1016/j.hroo.2026.02.030

**Published:** 2026-03-14

**Authors:** Angela W.C. Lee, Marina Strocchi, Alphonsus Liew, Sandra Howell, Felicity De Vere, Nadeev Wijesuriya, Ronak Rajani, Christopher Aldo Rinaldi, Steven Niederer

**Affiliations:** 1 National Heart and Lung Institute, Imperial College London, London, United Kingdom; 2 Cardiac Rhythm Management, Medtronic, London, United Kingdom; 3 Guy’s and St Thomas’ NHS Foundation Trust, London, United Kingdom; 4 King’s College London, St Thomas’ Hospital, London, United Kingdom; 5 Alan Turing Institute, London, United Kingdom

**Keywords:** Heart digital twins, CSP, conduction system pacing, Right ventricular pacing, Leadless pacemaker, LBBAP, Left bundle branch area pacing

## Abstract

**Background:**

Conduction system pacing with leadless left bundle branch area pacing (LBBAP) is a promising application of leadless pacemakers (L-PMs). However, the mechanical impact of the L-PM on intracardiac structures may worsen tricuspid regurgitation and induce ventricular arrhythmia. The optimal site of leadless conduction system pacing also remains unclear.

**Objective:**

This study aimed to quantify device- and site-specific collision risks and identify the optimal site for leadless LBBAP using computational modeling.

**Methods:**

We conducted a modeling study assessing collision risks of contemporary (Micra TPS, Aveir AR, and Aveir VR) and future L-PM devices and exploring the optimal site for leadless LBBAP using cardiac computed tomography models from 10 patients with heart failure. Virtual L-PM implantation on the right ventricular (RV) septal wall was performed, and models of the RV free wall, papillary muscles, moderator band, and tricuspid valve structures enabled assessment of device–structure interactions. Computer simulations examined collision risk across devices of varying dimensions throughout the cardiac cycle.

**Results:**

Collision risk increased with device length and volume, primarily driven by RV wall interactions. Apical pacing sites carried a 73.6%–75.2% collision risk, and overall wall collisions increased by 3%–8% per 5 mm device length. Tricuspid valve and papillary muscle collisions depended on implant region, with the highest risks of collision in basal-inferoseptal (>70%) and mid-inferoseptal regions (>40%), respectively. Leadless LBBAP via the left anterior fascicle had the lowest collision rates.

**Conclusion:**

These findings support patient-specific device selection and careful septal targeting of leadless LBBAP to minimize complications, optimize physiological activation, and guide future L-PM designs.

## Graphical abstract

**Figure:**
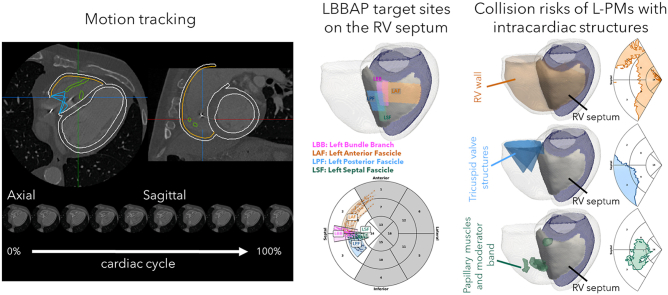

**Key Findings**

▪ Device length is the critical design parameter for intracardiac collision risk during leadless conduction system pacing, with collision risk increasing by 3%–8% per 5 mm increment (r = 0.997), primarily driven by right ventricular wall interactions. For contemporary leadless pacemakers, volume differences contributed minimal variation (<2%) at matched lengths.
▪ The left anterior fascicle region emerged as the optimal target for leadless left bundle branch area pacing among clinically feasible sites, offering the lowest combined collision risk (<53% at device lengths <35 mm) while maintaining appropriate mid-to-upper septal positioning for effective conduction system capture.
▪ Tricuspid valve (TV) collision risk was determined primarily by implant location rather than device design, with pacing sites >20 mm from the TV annulus substantially reducing TV collision risk across all device types.
▪ Substantial interpatient anatomic variability (standard deviation 15%–25% across regions) demonstrates that collision risk is highly anatomy dependent, supporting a patient-specific computational modeling approach to optimize device selection and implant site planning for leadless conduction system pacing.

## Introduction

Conventional apical right ventricular (RV) pacing is associated with ventricular dyssynchrony, leading to increased incidence of atrial fibrillation, heart failure (HF), pacing-induced cardiomyopathy (PICM), and mortality.^1^^,^^2^ Conduction system pacing (CSP) delivered by left bundle branch area pacing (LBBAP) may reduce or remove this dyssynchrony.^3^^,^^4^ CSP with LBBAP is presently delivered using transvenous pacemakers with the transvenous lead screwed deep into the interventricular septum. Transvenous leads are associated with higher rates of infection and tricuspid valve (TV) dysfunction.^5^ Leadless cardiac pacing using a pacemaker delivered to the RV is increasingly used in patients with bradyarrhythmias.^3^^,^^6^ Leadless pacemakers (L-PMs) have been found to reduce infection rates, with fewer major complications, shorter hospital stays, elimination of lead-related complications, and avoidance of pocket infections.^3^ However, they can still cause or worsen tricuspid regurgitation (TR) in 20%–40% of patients^7^^,^^8^ and have increased risks of perforation (0.8% vs 0.4%),^9^ and case reports suggest that L-PMs may cause arrhythmias via mechanical myocardial interaction.^10^^,^^11^ Presently, L-PMs deliver RV pacing, which may induce ventricular dyssynchrony and cause PICM, a complication occurring in 12% of patients with chronic RV pacing.^2^

The ability to deliver leadless CSP would represent a paradigm shift, but to date has been performed only acutely.^12^ Although acute CSP delivery using standalone RV-based L-PMs seems feasible, chronic leadless CSP remains untested. Key unanswered questions include optimal RV septal placement for CSP achievement and possible interactions with myocardial structures, RV endocardium, and TV apparatus. Moreover, it is unclear what device dimensions are optimal for RV implantation and which of the available devices would be best suited to deliver CSP.

Personalized computer models allow for patient-specific recommendations of pacing sites.^13^^,^^14^ Individual variation in the geometry and motion of the heart can affect the collision risk of varying L-PM lengths and designs. In previous work, left ventricular deformations were tracked in patients with HF, and the resulting cardiac motion was used to assess for collision risk of L-PMs in the left ventricle.^15^ In this modeling study, we extend this approach to the RV, investigating optimal device implant location in the RV septum to achieve CSP. Furthermore, we investigate how device dimensions affect the frequency of mechanical contact when devices are implanted to the RV septum for CSP using a systematic analysis of virtual implants across a cohort of HF patients to inform optimal device dimensions and location to achieve chronic CSP.

## Methods

### Ethics statement

Data were gathered as part of a clinical trial, REC number 14/WM/1069, approved by the West Midlands Coventry and Warwick ethics committee. Patients gave a written informed consent for imaging data to be used for research, and data were analyzed anonymously in accordance with the Declaration of Helsinki ethical guidelines.

### Clinical data

In an earlier research study, 24 patients with HF (New York Heart Association class 2–3) with existing transvenous RV leads indicated for cardiac resynchronization therapy upgrade were recruited.^16^ We identified a subcohort of 10 cases (100% male) where the RV papillary muscle (PM) and moderator band could be identified by an expert cardiac imaging researcher. These patients had dilated RVs (end-diastolic volume [EDV] 283 ± 38; end-systolic volume [ESV] 219 ± 45 mL vs EDV 163 ± 25 mL; ESV 57 ± 15 mL [healthy males]).^17^ Retrospectively gated cardiac computed tomography (CCT) scans of the heart throughout 10 phases (10% increment) of the cardiac cycle were acquired using a Philips Brilliance iCT scanner (Philips Healthcare, Amsterdam, The Netherlands) during RV pacing.

### Mesh generation and motion tracking

**Figure 1:**
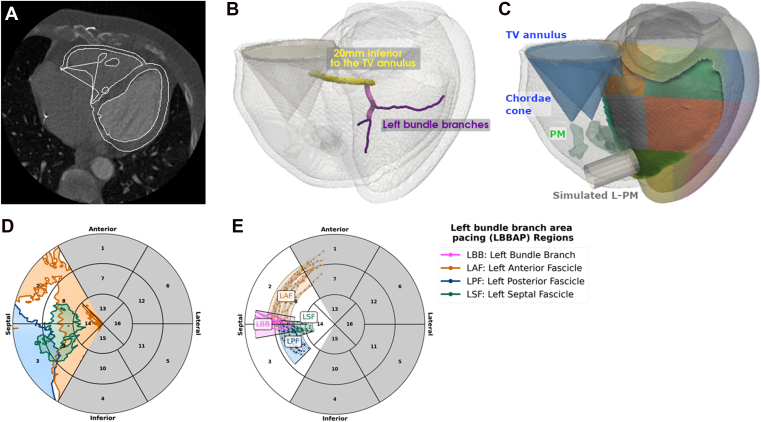
**A:** Cardiac computed tomography images were segmented and used to generate a (**B**) biventricular mesh, including the PMs, TV apparatus, and an estimation of the conduction system. **C:** American Heart Association regions were mapped onto the left ventricular epicardium and right ventricular (RV) septum. *Cylinders* representing the leadless pacemakers were virtually implanted across the RV septum surface. **D:** The >50% collision risk with the RV wall (*orange*), PM (*green*), and TV structures (*blue*). **E:** Left bundle branch area pacing regions were plotted. Non-RV septal regions are gray. L-PM = leadless pacemaker; PM = papillary muscles and moderator band; TV = tricuspid valve.

In a previous study, patient-specific 4-chamber heart meshes were generated and were made publicly available (https://zenodo.org/records/3890034).^16^ The mesh generation methods are described here in brief, and for further details, please refer to Strocchi et al.^16^ CCT images at LV end diastole were automatically segmented using the Siemens Axseg software to label the LV myocardium and blood pools for each of the 10 patients at the end-systolic phase (Figure 1).^18^ The RV blood pool was dilated by 3.5 mm to estimate the RV wall based on literature values,^19^ given that the thin RV free wall had insufficient contrast for direct segmentation in CT images. The TV plane was modeled as a 2-mm-thick plane between the right atria and RV blood pools.^16^

The publicly available meshes included an estimation of the proximal left bundle branch (LBB), left anterior fascicle (LAF), left posterior fascicle (LPF), and left septal fascicle (LSF) branches for all patients, defined using a publicly available tool (https://github.com/fsahli/fractal-tree) with seed points based on the universal ventricular coordinates of the heart^16^^,^^20^

RV intracardiac structures comprising the moderator band and anterior, inferior, and septal PM structures were not included in the previous automatic segmentation. In the present study, a gradient edge detection filter was applied to the LV end-diastolic image for each patient (not publicly available) for manual segmentation of the RV PMs by an expert cardiac imaging researcher. The basal-most point of each PM defined the chordae tendineae attachment point (Figure 1), from which a cone was projected to the TV annulus to define the volume enclosing the TV structures (annulus, leaflets, and chordae tendineae). Gmsh (http://gmsh.info) was used to generate finite element meshes from these geometries.^13^ In clinical studies, it has been found that pacing within regions <20 mm of the TV plane was associated with significantly higher rates of TV dysfunction; therefore, for each patient case, the region within 20 mm of the TV plane was defined for the meshes in the present study.^21^^,^^22^ Motion estimated from retrospectively gated CT using CemrgApp (https://cemrgapp.com) was then applied to the anatomic model to estimate wall and intracardiac structure motion throughout 10 phases of the cardiac cycle.^15^

### Collision risk assessment framework

Cylinders, representing the L-PM devices, were virtually implanted at each septal wall node, oriented perpendicular to the surface normal. We assessed whether the devices would collide with the RV intracardiac structures throughout the 10 frames of the cardiac cycle for each patient, using a previously published collision detection framework.^15^ Any collision was considered significant, with no minimum overlap threshold applied. Points within 10 mm of the attachment site or on the septum itself were excluded to avoid self-intersection. For each patient, collision was evaluated at every septal node across all time frames; a site was classified as positive if collision occurred in at least 1 time frame. A site was classified as positive for combined collision risk if it exhibited collision with any 1 or more of the RV wall, TV, or PM structures. To enable comparison across patients, patient-specific septal meshes were mapped onto a standardized coordinate grid, and the number of patients with collision at each location was summed. Regional statistics were then calculated for each American Heart Association (AHA) segment, with collision prevalence reported as the mean and standard deviation across patients expressed as a percentage.

**Figure 2:**
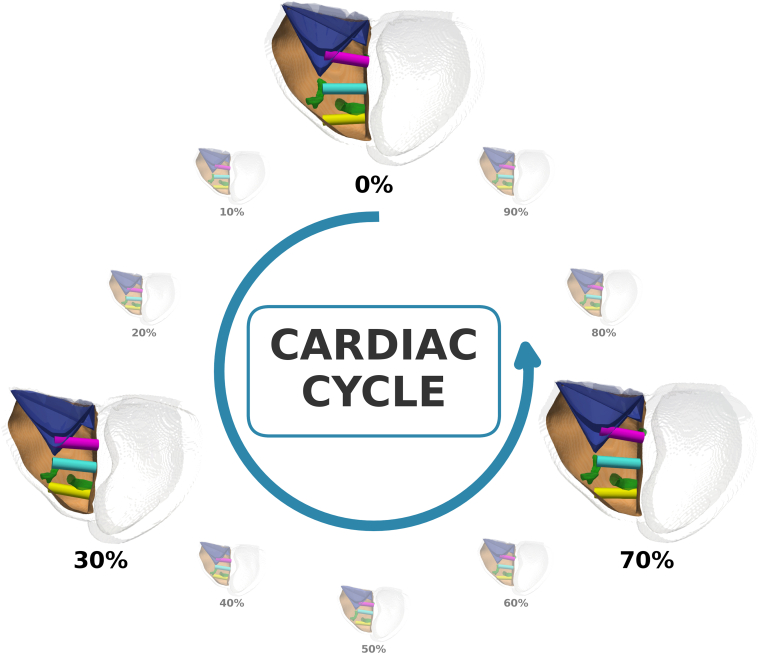
Leadless pacemakers (L-PMs) were virtually implanted across the right ventricular (RV) septal wall, and the deformations of the cardiac tissue were tracked. Collision with the RV intracardiac structures at any point throughout the 10 phases of the cardiac cycle was assessed for each implant location for each patient. 3 representative implant locations of the Micra TPS on a patient case are shown here. *Top:* (*pink*) L-PM collides with the tricuspid valve apparatus. *Middle:* (*blue*) L-PM does not collide with the intracardiac structures. *Bottom:* (*yellow*) L-PM collides with the RV free wall and the papillary muscles throughout the cardiac cycle.

This method uses a validated motion-tracking approach^23^ within a collision risk assessment framework verified in the left ventricle,^15^ which was used to assess collision risk in the RV. L-PMs were modeled as cylinders and oriented perpendicular to the RV septal implant site (Figure 1C). We tracked the motion of the RV endocardium, intracardiac structures, and L-PMs and assessed collision of the pacemakers with the (1) RV endocardium, (2) PM, and (3) TV structures (Figure 2). AHA regions were defined on the LV epicardium (coinciding with the RV septal region) using landmarks at the apex, base, and RV attachment points,^14^ and the collision risks of pacing from the RV septum were plotted (Figure 1D). LBBAP regions spanning the locations of the estimated LBB and the left fascicle branches (LAF, LPF, and LSF) in the 10 cases were estimated (Figure 1E).

### L-PM dimensions

L-PMs presently on the market have varying lengths from 25.9 mm (Micra TPS) and 32.2 mm (Aveir AR) to 38 mm (Aveir VR). A bespoke L-PM has been used for acute CSP in an investigational study,^12^ but has not been chronically implanted and is not available for clinical use. The dimensions of this investigational device are the same as the Aveir AR (32 mm). Beyond existing L-PM dimensions, we simulated collision probability for feasible designs with lengths of 15–45 mm (5 mm increments) while maintaining contemporary L-PM volumes to preserve battery capacity.

### Statistical analysis

Statistical analyses were conducted using Python (version 3.11, statsmodels and scipy packages). First, the collision risk associated with state-of-the-art device dimensions was analyzed using a mixed-effects model with region as a random intercept and device type as a fixed effect. Analysis of variance (ANOVA) and pairwise *t* tests were used for post hoc comparisons, stratified by AHA or LBBAP region.

Second, to evaluate the collision risk of feasible devices with varying lead lengths, a separate mixed-effects model was fitted, again with region as a random intercept, but with device type and lead length as fixed effects. 2-way ANOVA by region and multiple pairwise comparisons explored interaction effects.

Third, to compare the relationship between collision risk and L-PM length across different collision fields (RV, TV, PM, and combined), linear regression models were fitted for each field, and their slopes were compared. The interaction between collision risk category and device length was assessed using 2-way ANOVA, with a grouped analysis comparing the RV collision field with the TV and PM fields combined.

Pairwise comparisons were performed using *t* tests with Bonferroni correction. Statistical significance was set at *P* < .05.

## Results

In this section, we present the results of predicting the probability of collision between the L-PMs and the RV endocardium or RV intracardiac structures.

### Existing devices

**Figure 3:**
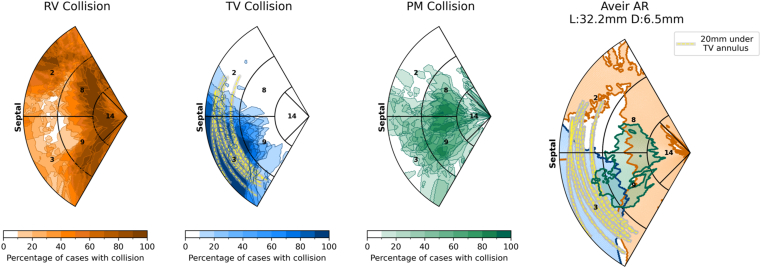
Probability maps of the likelihood of collision of the Aveir AR leadless pacemaker with the intracardiac structures (RV endocardium, PMs, and TV structures pacing at sites across the RV septum). The Aveir AR plot shows the RV septal pacing regions where more than 50% of cases would have collision with the RV (*orange*), TV (*blue*), or PM intracardiac structures (*green*). *Lines* denoting 20 mm below the TV annulus (*yellow dashed line*) for all 10 cases are shown. PM = papillary muscles and moderator band; RV = right ventricular; TV = tricuspid valve.

The collision results for each pacing site on the septum were interpolated and visualized on the RV septum AHA map for the 3 categories of intracardiac structures (RV, PM, and TV) and regions with >50% risk of collision with any structure. The results of the 10 patients are presented in Table 1, and the results for the Aveir AR (dimensions of the device used to deliver acute leadless CSP)^12^ are presented in Figure 3.^12^

**Table 1:** Patient demographics

Patient	Age	Sex	NYHA class	RV ESV (mL)	RV EDV (mL)	RVEF (%)	CT resolution (mm × mm × mm)
1	83	M	2	228	303	25	0.44 × 0.44 × 1.0
2	67	M	3	272	344	21	0.49 × 0.49 × 0.4
3	50	M	3	237	324	27	0.38 × 0.38 × 0.4
4	85	M	3	216	235	8	0.49 × 0.49 × 0.4
5	38	M	2	231	311	26	0.41 × 0.41 × 0.4
6	79	M	2	186	217	14	0.43 × 0.43 × 0.5
7	41	M	3	220	285	23	0.39 × 0.39 × 0.5
8	76	M	2	219	283	23	0.40 × 0.40 × 0.5
9	76	M	3	184	276	33	0.45 × 0.45 × 0.5
10	72	M	3	200	252	21	0.37 × 0.37 × 0.5
	67 ± 17			219 ± 45	283 ± 38	22 ± 7	

CT = computed tomography; EDV = end-diastolic volume; ESV = end-systolic volume; M = male; NYHA = New York Heart Association; RV = right ventricular; RVEF = right ventricular ejection fraction.

A mixed-effects model confirmed that the differences in overall collision risks between the devices were statistically significant (*P* < .0001). Micra TPS showed significantly fewer collisions than Aveir AR and VR across all AHA regions, whereas Aveir AR had lower collision risks than Aveir VR in nonapical areas (*P* < .0001). Pacing from the RV apex showed no significant difference between AR and VR devices (*P* = .385), with all 3 L-PMs having comparable overall collision risks (73.6%–75.2%) (Table 2). Detailed structure-specific collision risks are presented in Supplemental Section 1.

**Table 2:** The combined collision risk of current L-PM designs when pacing from AHA regions on the RV septum with intracardiac structures

	AHA regions					All RV septal regions
	2	3	8	9	14	
Combined collision risk						
Aveir VR	79.9 ± 15.9	94.4 ± 8.5	88.5 ± 14.8	91.6 ± 10.5	75.2 ± 24.1	85.0 ± 18.2
Aveir AR	54.6 ± 19.5	93.6 ± 9.3	78.3 ± 21.1	86.4 ± 12.0	74.9 ± 24.4	77.4 ± 22.5
Micra TPS	30.8 ± 21.2	91.1 ± 12.0	65.3 ± 26.4	81.3 ± 13.4	73.6 ± 23.9	68.9 ± 28.5

AHA = American Heart Association; L-PM = leadless pacemaker; RV = right ventricular.

### Feasible devices

**Figure 4:**
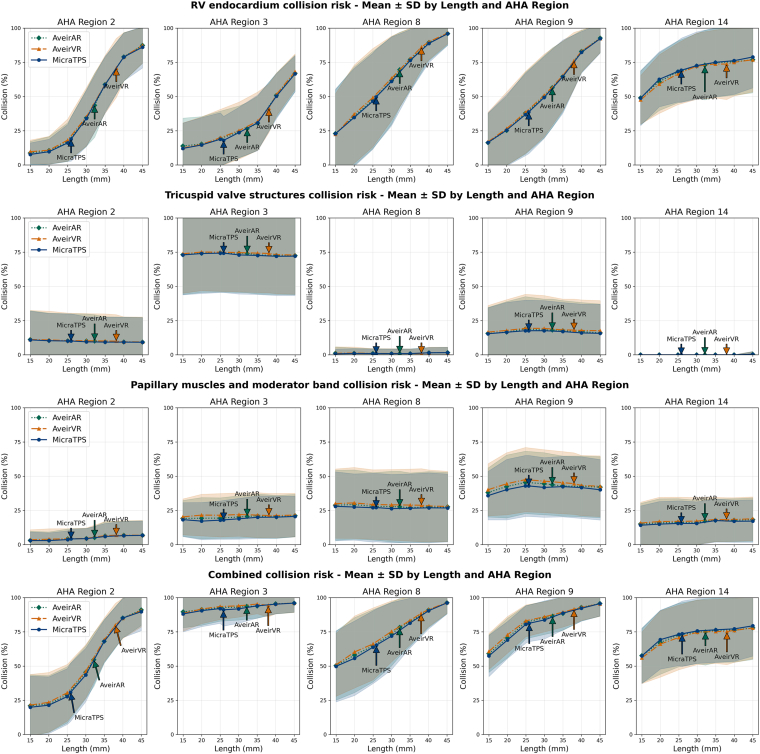
Plots of the collision risks with leadless pacemaker designs at different lengths within the RV septal AHA regions. The collision risks with the RV wall (top row), tricuspid valve structures (second row), papillary muscles and moderator band structures (third row), and the combined collision risk (bottom row) are presented. AHA = American Heart Association; RV = right ventricular; SD = standard deviation.

**Figure 5:**
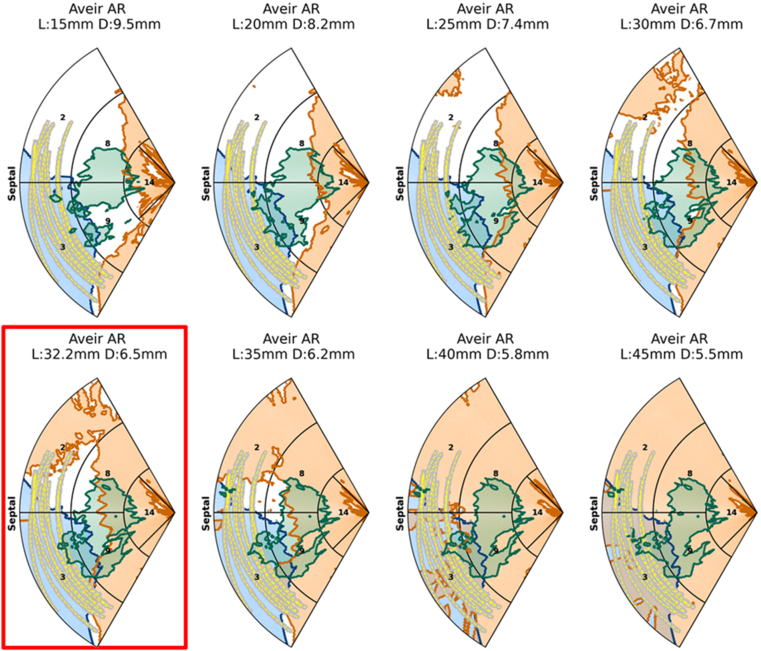
The combined collision risk throughout the cardiac cycle for 10 patients at different lengths of leadless pacemakers based on the Aveir AR device volumes plotted on the right ventricular septal American Heart Association regions. The results for the initial dimensions of the Aveir AR are boxed in *red*. Implantation areas where the leadless pacemaker would collide with the tricuspid valve apparatus (*blue*), papillary muscles and moderator band (*green*), and the right ventricular wall (*orange*) in >50% of cases and *yellow dashed lines* representing 20 mm under the tricuspid valve annulus are shown.

We tested feasible cylinder designs for all 3 L-PMs with lengths of 15–45 mm (5 mm increments) and corresponding diameters of 5.1–10.4 mm while preserving device volume (Figures 4 and 5). Overall collision risk increased with device length (+3%–8% per 5 mm increment; *P* < .0001), with this relationship consistent across all 3 device volumes (Figure 4). RV wall collision was the primary driver of length-dependent collision risk, showing significantly stronger length relationships than TV or PM collisions (detailed decomposition analysis in Supplemental Section 2). Regional analysis revealed that the basal-anteroseptal region had lower risks at shorter lengths, whereas the basal-inferoseptal region maintained consistently high collision risks across all lengths (detailed regional statistics in Supplemental Section 2).

### Optimal site for leadless CSP

**Figure 6:**
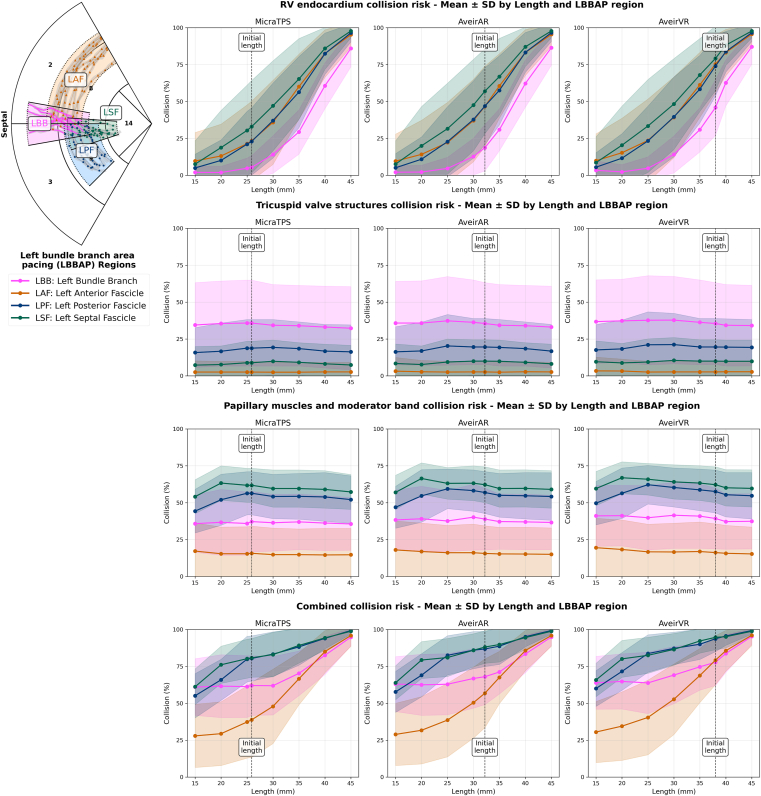
Plots of the collision risks with leadless pacemaker designs (Micra TPS [left], Aveir AR [middle], and Aveir VR [right]) at different lengths within the LBBAP regions. LBBAP = left bundle branch area pacing; RV = right ventricular; SD = standard deviation.

We identified 4 convex regions spanning our estimations of the LBBAP regions: LBB, LAF, LPF, and LSF areas. We evaluated the proportion of cases where these can be targeted as a function of device length (Figure 6).

ANOVA tests and post hoc pairwise comparisons found significant differences (*P* < .0001) in the collision risks between the L-PM devices with LBBAP, with Micra associated with lowest (LBB 62.0% ± 20.3%; LAF 38.8% ± 25.4%; LPF 80.4% ± 15.3%; LSF 80.8% ± 13.1%), Aveir AR medium (68.0% ± 18.9%, 56.8% ± 23.3%, 86.8% ± 11.9%, 88.1% ± 11.0%), and Aveir VR highest collision risks (77.8% ± 15%, 79.2% ± 16.6%, 93.7% ± 8.7%, 94.7% ± 8.4%), respectively.

We found strong length dependence for the RV wall collision and combined collision risks for all CSP regions, with shorter devices (<35 mm) having lower chances (<40%) of affecting the RV wall. We also found that the proximal LBB, situated higher in the RV septum, was associated with lower RV wall collisions, but more likely to collide with the TV apparatus than pacing via the more distal fascicle branches. At shorter device lengths (<35 mm), the LAF branch was associated with the lowest risks of overall collisions (<53%). For LPF, at shorter device lengths (<35 mm), the overall risk of collisions (55.1%–87.2%) was predominantly from PM (44.3%–62.2%), rather than RV wall (5.0%–39.6%) or TV apparatus collisions (15.8%–21.2%). A similar pattern was observed for LSF pacing collision risks at lengths of <35 mm (overall 61.2%–86.5%; PM 54.1%–66.9%; RV wall 7.6%–48.3%; TV apparatus 7.3%–10.5%).

We found that, across the 3 device volumes, with a length of 40 mm, only 16.3%–17.5%, 14.1%–14.7%, 4.4%–6.0%, and 4.9%–5.7% of the regional areas across the patient cases were collision-free when pacing from LBB, LAF, LPF, and LSF, respectively; this improved to 36.2%–38.7%, 63.0%–66.1%, 16.3%–20.1%, and 19.9%–23.9% at device lengths of 25 mm.

## Discussion

Using in silico modeling, we assessed the collision risks for existing and future L-PM designs to achieve CSP when pacing from various locations of the RV septum in LBBAP regions. Minimizing collision risk with intracardiac structures is crucial, given that mechanical impact can cause ventricular ectopic beats and induce arrhythmias^10^^,^^11^^,^^24^ and can cause or worsen TR through valve leaflet collision or chordae tendineae entanglement.^7^^,^^8^ Our modeling framework distinguishes collision risk by intracardiac structure, which may inform future L-PM designs based on the clinical significance of different collision types. It is important to note that although our model identifies possible anatomic risks, the reported incidence of clinically significant device-collision complications in large-scale trials and real-world registries for state-of-the-art L-PMs remains rare.^25^

### RV wall collision risk

In our simulations, we assessed the risk of collision with the RV wall throughout the cardiac cycle. Our simulations found high RV wall collision risks (>69%), especially at the apex. We found that the RV outflow tract (RVOT) (anterior section of AHA region 2) showed highly length-dependent collision risk, with low risk for the shortest Micra TPS (30.8% ± 21.1%), moderate for the Aveir AR (54.6% ± 19.5%), and high for the longest Aveir VR (79.9% ± 15.9%), indicating that although the RVOT is a good site to pace for the Micra TPS, this may not be the case for the Aveir VR.

RV apical pacing has also been found to be associated with increased risks of atrial fibrillation, PICM, and ventricular arrhythmia risk.^1^^,^^2^ Nonapical pacing sites, such as RV septal and RVOT pacing sites, have been found to be better at preserving cardiac function with lower PICM risks.^26^^,^^27^

### TV regurgitation risk

Consistent with previous studies, the highest TV collision risk occurred when implanting below the TV annulus, in the basal-inferoseptal region (>70%), particularly within 20 mm of the TV annulus.^21^^,^^22^ Adjacent regions showed low collision risk (regions 2 and 9 <20%), with minimal risk in apical and mid-anteroseptal regions (<1%). Importantly, collision risk with TV structures showed no substantial differences between device lengths or designs within each AHA region, indicating that pacing location selection (>20 mm distal from the model approximation of the TV annulus plane) is the primary mitigating factor for TR risk reduction. This distance threshold is based on mechanical collision avoidance rather than optimal electrophysiological positioning for LBBAP.

### Optimal site for CSP

CSP via LBBAP represents a promising strategy for achieving more physiological cardiac activation through earlier Purkinje network recruitment.^28^ Studies have found that although the LAF is long and thin, the LPF is shorter and fan-like in shape.^29^^,^^30^ Initial studies predominantly used LPF as the primary LBBAP site, given its anatomic accessibility and position as the main continuation of the LBB.^29^^,^^31^^,^^32^ However, the multicenter MELOS observational study (n = 2355) found a more balanced distribution of pacing locations across the left fascicles branches: LAF (24.8%), LPF (35.6%), and LSF (39.6%) rather than at the proximal LBB, reflecting both anatomic accessibility and practical considerations in achieving effective conduction system capture.^33^ This clinical reality informed our collision risk analysis across all LBBAP regions to identify optimal sites balancing mechanical safety with feasible CSP delivery.

A key finding of our patient-specific modeling approach is that optimal LBBAP site selection may vary substantially between individuals based on their unique cardiac anatomy. The LAF region, positioned in the mid-to-upper septum, balances mechanical collision avoidance (including >20 mm distance from TV annulus) with appropriate anatomic positioning for effective CSP, avoiding more distal/apical sites. Our analysis of the collision risks in each of the LBBAP regions found that, at lengths of <35 mm, the LAF branch coincides with low collision risk areas, whereas most of the collision risks for LPF pacing come from impact on the PMs and moderator bands, structures that vary among individuals. This suggests that patient-specific modeling could guide individualized device selection and implantation site planning, optimizing the balance between effective LBBAP capture and collision avoidance for each patient. Although achieving this level of personalization may require additional mapping or image-based guidance systems, our framework demonstrates the feasibility of such an approach. In addition, the patterns observed across our cohort may inform future device development for broader populations.

### Implications for device design

As expected, our analysis confirmed numerically an inverse relationship between device length and collision risk, with shorter devices having reduced collision risks. The shortest length (15 mm) achieved low RV wall collision risk (<20%) across all regions but required radii of 8.8–10.3 mm. However, catheter delivery limits device diameter, given that larger diameters require larger catheters, increasing vascular complication risks. Devices presently on the market demonstrate a clear volume-capacity trade-off: the smallest device (Micra, 1.0 cm^3^) has the lowest capacity (142 mAh), whereas larger devices (Aveir VR/AR, 1.4 cm^3^) house greater capacity (174–241 mAh).^34^^,^^35^ However, the Micra’s capacity is clinically sufficient, supporting an estimated longevity of more than 10 years, which establishes a validated benchmark for adequate chronic therapy.^36^

### Patient population considerations

Our study modeled hearts from male patients with HF with dilated RVs (EDV 283 ± 38 mL; ESV 219 ± 45 mL). Given that female hearts are generally smaller and the RVs were dilated within the HF cohort, our cohort selection provided an upper bound on the possible size of devices that can be implanted without interacting with cardiac structures. Normal RV dimensions are substantially smaller (healthy males, EDV 163 ± 25 mL, ESV 57 ± 15 mL; healthy females, EDV 126 ± 21 mL, ESV 43 ± 13 mL).^17^

Patients indicated for L-PM implantation, such as those with bradycardia, may have smaller RV dimensions that increase collision risk, and given that RV endocardial collisions were the primary determinant, our findings are likely accentuated in these patients. For patients with dilated RVs similar to our cohort, devices <35 mm in length are recommended to minimize RV wall collision risk (<40%). However, substantial interpatient anatomic variability (standard deviation 15%–25% across regions) indicates collision risk is highly anatomy dependent. Development of predictive formulas correcting for individual anatomies would require larger datasets, including females and patients with bradycardia, and represents a future direction of this work.

### Limitations

Several limitations warrant consideration when interpreting these findings. First, cardiac motion modeling was derived from preprocedural cine CCT data during RV apical pacing in patients with LBB block and HF. This dyssynchronous contraction pattern may not accurately represent myocardial deformation with nonapical pacing. In addition, our HF cohort exhibited dilated RV morphology and likely reduced radial contractility, which may have influenced the observed superiority of LBBAP via LAF. These findings may not be generalizable to patients with structurally normal hearts, and future studies in non-HF populations are needed to validate these results.

Second, the model assumes that the devices are perpendicular to the RV septum, and the device position in the ventricle is not static and moves with the RV septum throughout the cardiac cycle, and thus, estimates are likely to be overestimated compared with real-world variability in device orientation, angulation, and insertion depth, especially for LBBAP. In Supplemental Section 3, we tested the sensitivity of our models to device angulation. Although sampling orientations within a ±5° range reduced simulated regional risk by 1%–9%, the identified high-risk regions and relationships with device length remained consistent, supporting the robustness of our primary findings.

Third, our collision analysis modeled cardiac tissues as rigid structures and did not account for tissue deformability or compliance. In reality, moderate lead-tissue overlap may be accommodated through myocardial stretching or displacement without clinical consequence, and therefore, our binary collision metric may overestimate risk by not distinguishing between minor tissue contact and clinically significant mechanical interference. Future work could explore tissue compliance and quantify tolerable overlap thresholds as an additional axis of risk assessment.

Fourth, although our patient-specific models incorporated personalized cardiac geometry, wall motion, and intracardiac structures, conduction system locations were estimated using a standardized fractal tree algorithm rather than patient-specific reconstruction. Recent computational approaches have demonstrated the feasibility of reconstructing patient-specific His-Purkinje networks by integrating electroanatomic mapping data with cardiac imaging.^37^ However, identifying true patient-specific Purkinje networks remains a heavily ill-posed inverse problem, with multiple network configurations capable of reproducing the same activation patterns. In addition, ground truth human Purkinje network reconstructions for validation remain scarce,^38^, ^39^, ^40^ and therefore, such computationally derived networks should be interpreted as functional approximations rather than anatomically accurate representations. Further validation against ex vivo micro-CT and histologic reconstruction will be required before patient-specific conduction system reconstruction can be used to enhance the precision of personalized collision risk predictions in future studies. Furthermore, in our models, we have estimated the conduction system and modeled it as lines for each patient; however, anatomic studies have described the LPF as short and broad.^30^ Although our model may underestimate the LPF extent, collision constraints from the location of these structures remain unchanged.

Fifth, the RV PMs and the moderator band were often challenging to visualize. Manual segmentation was feasible only in cases with adequate image quality (image quality metrics are described in Supplemental Section 4) to identify the PMs and moderator band, underscoring the need for future assessments of interobserver variability or CCT imaging protocols optimized for RV structures. The cohort used in our study that had sufficient RV contrast, allowing for PM and moderator band definition, was all male. Given that female hearts are generally smaller, they would likely experience higher collision rates with L-PM devices, and our findings may underestimate collision risk in female patients. There was insufficient imaging contrast to directly identify the RV wall and the TV plane in the images. Therefore, our modeling approach approximated the TV as a plane at the interface between the dilated RV and right atrial blood pools. The RV wall thickness was estimated at 3.5 mm through uniform dilation of the blood pool. Although this approach provides a reasonable approximation for our collision risk assessment, it does not capture potential regional variations in wall thickness. However, given that collision risk is primarily determined by chamber geometry and motion rather than precise wall thickness, this simplification is unlikely to substantially affect our main findings.

Finally, the simulations assumed unrestricted feasibility of implantation along the RV septum without accounting for procedural considerations such as venous access, delivery sheath maneuverability, device stability, or septal thickness heterogeneity.

### Clinical implications and future directions

The present findings have several implications for device selection and implantation strategy. Among systems available for clinical use, the Micra TPS demonstrated the most favorable overall collision profile, with the lowest nonapical RV wall collision risk (<60%) (Supplemental Table 1). The Aveir AR exhibited higher overall RV collision risk than Micra TPS but maintained relatively low risk within the RVOT and basal septal regions. In contrast, the Aveir VR was associated with high collision risk (>75%) across all regions. At present, the only device that has been used to deliver acute leadless CSP is based on the Aveir AR that uses a fixation helix with an extended pacing/sensing electrode; however, this device is not commercially available.^12^ The Micra has a different fixation mechanism, which would not allow CSP unless there was a significant change to its fixation design. The contemporary generation of L-PMs is likely to have significant limitations in terms of chronic CSP delivery owing to their size. Alternative approaches for leadless CSP delivery, such as endocardial left ventricular pacing systems that access the septum from the LV side,^41^ may mitigate the RV pacing-based collision constraints identified in this study.

Our study suggests that optimal pacing sites for leadless CSP should prioritize avoidance of high-risk regions, particularly in the basal-inferoseptal region, where TV collision risk exceeded 70%. LBBAP via the LAF may be more feasible, combining a favorable safety profile with preservation of physiological ventricular activation. Device length emerged as an important determinant of collision risk. This may inform the development of shorter devices, which are associated with reduced collision risk, but this must be balanced against practical considerations of device longevity and delivery system constraints.

## Conclusion

This computational modeling study provides novel quantification of device- and site-specific determinants of RV collision risk types during leadless CSP implantation, providing a tool for future device design and allowing for patient-specific collision risk assessment to optimize patient-specific care. Collision risk increased with device length. The Micra TPS exhibited the lowest overall collision profile, whereas longer devices required careful regional targeting to mitigate risk. Importantly, TV collision was determined primarily by implant location rather than device design, emphasizing the importance of maintaining a distance of >20 mm from the annulus. Within the clinically feasible LBBAP target regions analyzed, the LAF region demonstrated the lowest predicted mechanical collision risk for device lengths of <35 mm. These results quantify mechanical constraints relevant to leadless CSP design and placement and should be integrated with electrophysiological and clinical site-selection considerations.

These observations support a personalized approach to L-PM implantation to achieve CSP, integrating patient-specific anatomy, device characteristics, and pacing site selection to optimize outcomes while minimizing complications.

## Declaration of generative AI and AI-assisted technologies in the writing process

The authors used ChatGPT and Claude to improve the readability of their work. After using these tools, the authors reviewed and edited the content as needed and take full responsibility for the content of the publication.

## Disclosures

N.W., A.L., F.d.V., and S.H. are supported by the Wellcome/Engineering and Physical Sciences Research Council Centre for Medical Engineering (WT203148/Z/16/Z). N.W. receives funding from the British Heart Foundation (FS/CRTF/22/24362). C.A.R. receives research funding and/or consultation fees from Abbott, Medtronic, Boston Scientific, Philips, and EBR Systems. The other authors have no conflicts of interest to disclose.

## Footnotes

Appendix Supplementary data associated with this article can be found in the online version at https://doi.org/10.1016/j.hroo.2026.02.030.
