# Supplementary material for: Computational analysis of intracardiac collision risk and optimal site for right ventricular leadless left bundle branch area pacing: A simulation study
Source: Heart Rhythm O2. 2026 Mar 14;7(5):947–58. doi: 10.1016/j.hroo.2026.02.030 (PMC13198349; doi:10.1016/j.hroo.2026.02.030)
Supplement: Supplementary File [file mmc1.docx]

**Supplement:**

**S1. Detailed Collision Risk Analysis by Structure Type**

Analysis of each of the separate categories of collision indicated significant differences between the L-PMs for the RV wall and PM structures (p<0.0001), and no difference for the TV collision risk. We found comparable PM structure collision risks, with <5% difference across the different AHA regions for the 3 L-PMs. Highest collision risks were in the mid-inferoseptal region for PMs (43-45%) and basal-inferoseptal region for TV (74%) (Table S2).

The RV endocardium collision risk was significantly and substantially different across AHA regions for the 3 devices, with higher risk corresponding to longer length of the device (p<0.0001). The exception was in the apex, with no statistically significant difference between the Aveir AR and VR devices (p=0.064), and while the Micra TPS was significantly different (p<0.0001), the RV collision risk was comparable across the devices (<5% difference).

**Table S1:** The collision risk of contemporary L-PM designs when pacing from AHA regions on the RV septum against intracardiac structures (RV endocardium, Papillary muscles and moderator band; Tricuspid valve structures.)

|  | AHA regions | | | | |  |
| --- | --- | --- | --- | --- | --- | --- |
|  | 2 | 3 | 8 | 9 | 14 | All RV septal regions |
| Tricuspid valve collision risk | | | | | |  |
| Aveir VR | 10.0 ± 19.3 | 74.1 ± 28.8 | 0.9 ± 3.4 | 18.1 ± 22.6 | 0.0 ± 0.0 | 18.7 + 32.1 |
| Aveir AR | 10.0 ± 19.3 | 74.1 ± 28.8 | 0.9 ± 3.4 | 18.1 ± 22.6 | 0.0 ± 0.0 | 18.7 + 32.1 |
| Micra TPS | 10.1 ± 19.8 | 74.3 ± 28.8 | 0.9 ± 3.4 | 17.6 ± 22.3 | 0.0 ± 0.0 | 18.7 + 32.2 |
| Papillary muscles and moderator band collision risk | | | | | |  |
| Aveir VR | 6.9 ± 10.4 | 21.3 ± 16.1 | 28.8 ± 26.7 | 44.6 ± 22.5 | 18.3 ± 16.0 | 23.6 ± 22.5 |
| Aveir AR | 5.2 ± 8.2 | 20.6 ± 15.5 | 27.6 ± 26.0 | 43.8 ± 22.3 | 17.4 ± 16.0 | 22.5 ± 22.2 |
| Micra TPS | 4.2 ± 7.3 | 18.1 ± 14.2 | 27.1 ± 25.0 | 42.9 ± 22.3 | 15.5 ± 16.5 | 21.2 ± 22.0 |
| RV wall collision risk | | | | | | |
| Aveir VR | 70.9±21.6 | 41.4±17.7 | 86.2±16.3 | 76.2±20.7 | 73.4±25.9 | 70.2±25.6 |
| Aveir AR | 43.6±21.6 | 26.5±19.1 | 69.6±27.4 | 56.5±29.0 | 73.0±25.4 | 55.6±30.5 |
| Micra TPS | 18.9±16.1 | 18.0±17.5 | 49.7±34.7 | 38.7±30.4 | 69.2±23.5 | 41.6±32.5 |

**S2. Statistical Analysis of Length-Dependent Collision Risk**

Mixed-effects modeling revealed significant main effects for both device type and lead length (p<0.0005). We found that overall collision risk correlated with device volume: Micra-TPS based designs (smallest volume) showed significantly lower collision values than Aveir AR based designs, while Aveir VR based designs (largest volume) had higher collision risks.

The overall collision risk had a strong length dependence, increasing steadily from 15-45mm (+3-8% per 5mm increment, p < 0.0001) (Figure 4). Within-device ANOVA confirmed strong linear relationships between length and collision risk for all devices (p < 0.0001), with all pairwise length comparisons significant. Between-device comparisons found no substantial differences between the collision risk at the same length (<2%). Mixed-effects modeling revealed significant regional variations, where the basal-anteroseptal region had lower risks at shorter lengths, while the basal-inferoseptal region had consistently high collision risks.

Decomposition analysis found that RV collision is the primary driver of the length-dependent collision risk. RV collision demonstrated the strongest length relationship, with slope magnitudes 15.6-fold and 23.2-fold greater than TV Collision and PM Collision, respectively. RV Collision showed the highest correlation with the combined pattern (r=0.997, p<0.001) compared to PM Collision (r=0.936, p=0.002) and TV Collision (r=-0.369, p=0.416). Two-way ANOVA confirmed significant collision field × length interaction (p<0.0001), with RV Collision responding differently to length changes than TV Collision and PM Collision combined (p<0.0001).

**S3. Sensitivity Analysis on the Impact of Device Angulation on Collision Risk**

The analysis in the manuscript assumed devices remained perpendicular to the RV septum. In this supplement section we performed a sensitivity analysis to test the impact of device angulation by sampling ten orientations within a ±5° rotation at each septal attachment point. A region was classified as collision-free if any sampled orientation avoided RV structures. This allowance for angulation reduced the simulated collision risk in each AHA/LBBAP region by 1–9% compared to the fixed perpendicular model (Tables S3 & S4). Despite this reduction, the relationships between device length and collision risk, as well as the identification of highest-risk regions, remained consistent with our primary findings (Figure S1).

A comprehensive simulation of all possible device angles at each septal location was not performed due to prohibitive computational cost. For each patient, collision risk was assessed at 1000-3000 septal points (dependent on mesh size) across 10 cardiac timepoints. The 5-degree angular sensitivity analysis required 9 additional simulations per septal point per timepoint. This sampling strategy achieved approximately 50% overlap between adjacent device simulations at the distal end of the cylinder for the Aveir VR device (the longest tested leadless pacemaker). Extending this approach while maintaining equivalent overlap density would require an additional 18 simulations per septal point for ±10° angulation, and approximately 1200 simulations per septal point to cover a full hemispherical range (±90°) for each cardiac timepoint in each patient.

**Table S2:** Effect of ±5° directional rotation on collision risk. Values represent the mean difference in collision percentage between cone rotation (±5°) and standard (0°) configurations across all device lengths tested. Positive values indicate increased collision risk with rotation.

# Data presented as Mean ± Standard Deviation (%) for the mean difference of AHA regions 2, 3, 8, 9, 14, and all the RV septal regions.

| Device | 2 | 3 | 8 | 9 | 14 | All Regions |
| --- | --- | --- | --- | --- | --- | --- |
| RV Collision | | | | | | |
| Aveir VR | -7.8 ± 1.8 | -6.5 ± 3.8 | -5.3 ± 1.7 | -5.9 ± 2.0 | -5.2 ± 0.5 | -6.1 ± 2.0 |
| Aveir AR | -7.3 ± 1.5 | -5.6 ± 3.3 | -5.6 ± 1.7 | -6.1 ± 1.9 | -5.9 ± 0.7 | -6.1 ± 1.8 |
| Micra TPS | -6.9 ± 1.6 | -5.6 ± 3.6 | -5.7 ± 1.7 | -6.1 ± 1.7 | -6.3 ± 0.5 | -6.1 ± 1.8 |
| TV Collision | | | | | | |
| Aveir VR | -1.4 ± 0.3 | -3.1 ± 0.5 | -0.5 ± 0.1 | -3.4 ± 0.5 | -0.1 ± 0.2 | -1.7 ± 0.3 |
| Aveir AR | -1.4 ± 0.2 | -3.3 ± 0.5 | -0.5 ± 0.1 | -3.3 ± 0.6 | -0.1 ± 0.2 | -1.7 ± 0.3 |
| Micra TPS | -1.4 ± 0.2 | -3.3 ± 0.6 | -0.4 ± 0.1 | -3.4 ± 0.8 | -0.1 ± 0.2 | -1.7 ± 0.4 |
| PM Collision | | | | | | |
| Aveir VR | -2.6 ± 1.3 | -2.9 ± 1.0 | -3.2 ± 0.5 | -6.7 ± 1.7 | -4.5 ± 1.5 | -4.0 ± 1.2 |
| Aveir AR | -2.2 ± 1.1 | -2.8 ± 0.9 | -3.2 ± 0.7 | -6.7 ± 1.7 | -4.1 ± 1.5 | -3.8 ± 1.2 |
| Micra TPS | -2.1 ± 1.0 | -2.6 ± 0.7 | -3.2 ± 0.7 | -6.7 ± 1.5 | -4.0 ± 1.4 | -3.7 ± 1.1 |
| Combined Collision | | | | | | |
| Aveir VR | -8.6 ± 2.2 | -2.9 ± 1.0 | -5.0 ± 1.4 | -5.1 ± 1.7 | -5.1 ± 0.4 | -5.3 ± 1.3 |
| Aveir AR | -8.3 ± 1.9 | -3.1 ± 0.7 | -5.3 ± 1.4 | -5.9 ± 1.6 | -5.7 ± 0.6 | -5.7 ± 1.2 |
| Micra TPS | -8.0 ± 1.7 | -3.5 ± 0.7 | -5.5 ± 1.5 | -6.5 ± 1.8 | -6.1 ± 0.7 | -5.9 ± 1.3 |

**Table S3:** Effect of ±5° directional rotation on collision risk for the left bundle branch area pacing areas. Values represent the mean difference in collision percentage between cone rotation (±5°) and standard (0°) configurations across all device lengths tested. Positive values indicate increased collision risk with rotation.

Data presented as Mean ± Standard Deviation (%) for the mean difference of the LBBAP regions.

| Device | Proximal Left Bundle Branch | | Left Anterior Fascicle | | Left Posterior Fascicle | | Left Septal Fascicle | |
| --- | --- | --- | --- | --- | --- | --- | --- | --- |
| RV Collision | | | | | | | |  |
| AveirAR | -5.1 ± 3.5 | -5.7 ± 2.6 | | -6.2 ± 3.1 | | -5.8 ± 2.8 | |  |
| AveirVR | -6.5 ± 4.3 | -5.6 ± 2.6 | | -6.2 ± 3.2 | | -5.9 ± 3.1 | |  |
| MicraTPS | -4.9 ± 3.9 | -5.3 ± 2.3 | | -5.9 ± 2.9 | | -5.5 ± 2.5 | |  |
| TV Collision | | | | | | | |  |
| AveirVR | -5.2 ± 0.5 | -0.8 ± 0.1 | | -4.8 ± 0.9 | | -2.5 ± 0.5 | |  |
| AveirAR | -5.1 ± 0.5 | -0.8 ± 0.1 | | -4.7 ± 1.0 | | -2.6 ± 0.5 | |  |
| MicraTPS | -5.1 ± 0.6 | -0.7 ± 0.1 | | -4.8 ± 1.2 | | -2.5 ± 0.6 | |  |
| PM Collision | | | | | | | |  |
| AveirVR | -6.0 ± 1.1 | -2.4 ± 0.4 | | -8.7 ± 2.4 | | -8.4 ± 1.5 | |  |
| AveirAR | -6.1 ± 1.0 | -2.2 ± 0.5 | | -9.0 ± 2.4 | | -8.8 ± 1.4 | |  |
| MicraTPS | -6.3 ± 0.9 | -2.2 ± 0.4 | | -9.1 ± 2.4 | | -9.3 ± 1.4 | |  |
| Combined Collision | | | | | | | |  |
| AveirVR | -6.4 ± 1.5 | -6.0 ± 2.2 | | -6.0 ± 2.5 | | -5.0 ± 2.5 | |  |
| AveirAR | -6.4 ± 1.4 | -6.2 ± 2.2 | | -6.8 ± 2.3 | | -5.8 ± 2.2 | |  |
| MicraTPS | -6.6 ± 1.5 | -6.0 ± 2.0 | | -7.5 ± 2.6 | | -6.7 ± 2.4 | |  |


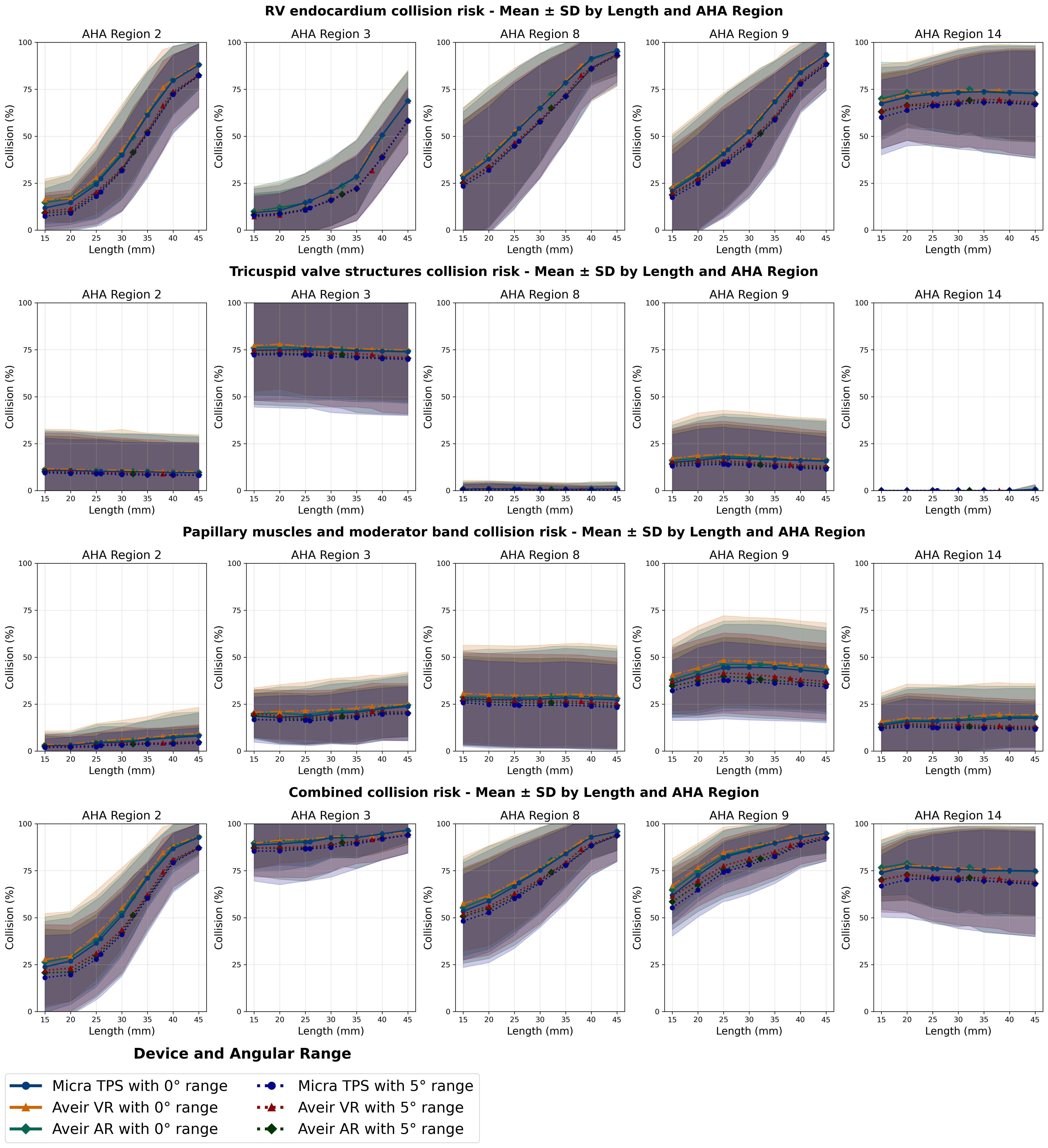


Figure S1: Plots of the collision risks for three L-PM designs across varying implant lengths and angular ranges within the RV septal AHA regions.

**S4. Image quality metrics for papillary muscle and moderator band identification from CCT images.**

Image quality was assessed for each scan to evaluate the visibility of right ventricular papillary muscles and the moderator band. Signal-to-noise ratio (SNR) and contrast-to-noise ratio (CNR) were computed for the papillary muscle regions relative to the adjacent RV blood pool. A gradient-based edge detection filter was applied to the RV blood pool to enhance structural boundaries prior to manual identification. Across the 10 successfully processed cases, SNR of the papillary muscles was 2.17 ± 0.56 (mean ± SD) and CNR between the papillary muscles and RV blood pool was 0.30 ± 0.35. These modest values are consistent with the known challenges of imaging small RV structures in CCT, where contrast enhancement is less robust than in the left ventricle. This variability motivated the use of a gradient-based edge detection approach, which yielded consistent performance (SNR 0.86 ± 0.04) across all cases regardless of the underlying intensity contrast. Note that gradient SNR is not directly comparable to intensity-based SNR, as it measures edge strength relative to noise in the derivative domain rather than signal magnitude. All papillary muscles and moderator bands were visually identifiable in every case by an expert cardiac imaging researcher. Per-scan metrics are reported in Supplementary Table S1.

**Table S4:** Image quality metrics for papillary muscle and moderator band identification from CCT images.

| **Case** | **PM Voxels** | **SNR (PM)** | **CNR (PM vs RV)** | **SNR (Gradient)** |
| --- | --- | --- | --- | --- |
| 01 | 62,141 | 1.994 | 0.361 | 0.806 |
| 02 | 167,103 | 2.188 | 0.057 | 0.875 |
| 03 | 132,284 | 1.337 | 1.132 | 0.863 |
| 04 | 189,016 | 2.597 | 0.003 | 0.897 |
| 05 | 149,317 | 3.096 | 0.148 | 0.856 |
| 06 | 124,400 | 2.492 | 0.089 | 0.877 |
| 07 | 141,176 | 1.136 | 0.716 | 0.899 |
| 08 | 135,664 | 2.258 | 0.149 | 0.799 |
| 09 | 107,787 | 2.597 | 0.186 | 0.863 |
| 10 | 89,605 | 2.049 | 0.222 | 0.808 |
| **Mean** | **129,849** | **2.174** | **0.306** | **0.854** |
| **SD** | **36,751** | **0.591** | **0.354** | **0.037** |
